# Reproducible In Vitro Tissue Culture Model to Study Basic Mechanisms of Calcific Aortic Valve Disease: Comparative Analysis to Valvular Interstitials Cells

**DOI:** 10.3390/biomedicines9050474

**Published:** 2021-04-26

**Authors:** Andreas Weber, Melissa Pfaff, Friederike Schöttler, Vera Schmidt, Artur Lichtenberg, Payam Akhyari

**Affiliations:** Department of Cardiac Surgery, Medical Faculty, University Hospital Düsseldorf, Heinrich-Heine-University Düsseldorf, 40225 Düsseldorf, Germany; andreas.weber@med.uni-duesseldorf.de (A.W.); melissa.pfaff@hhu.de (M.P.); friederike.schoettler@med.uni-duesseldorf.de (F.S.); vera.schmidt@med.uni-duesseldorf.de (V.S.); payam.akhyari@med.uni-duesseldorf.de (P.A.)

**Keywords:** aortic valve stenosis, calcific aortic valve disease, CAVD, calcification, degeneration

## Abstract

The hallmarks of calcific aortic valve disease (CAVD), an active and regulated process involving the creation of calcium nodules, lipoprotein accumulation, and chronic inflammation, are the significant changes that occur in the composition, organization, and mechanical properties of the extracellular matrix (ECM) of the aortic valve (AV). Most research regarding CAVD is based on experiments using two-dimensional (2D) cell culture or artificially created three-dimensional (3D) environments of valvular interstitial cells (VICs). Because the valvular ECM has a powerful influence in regulating pathological events, we developed an in vitro AV tissue culture model, which is more closely able to mimic natural conditions to study cellular responses underlying CAVD. AV leaflets, isolated from the hearts of 6–8-month-old sheep, were fixed with needles on silicon rubber rings to achieve passive tension and treated in vitro under pro-degenerative and pro-calcifying conditions. The degeneration of AV leaflets progressed over time, commencing with the first visible calcified domains after 14 d and winding up with the distinct formation of calcium nodules, heightened stiffness, and clear disruption of the ECM after 56 d. Both the expression of pro-degenerative genes and the myofibroblastic differentiation of VICs were altered in AV leaflets compared to that in VIC cultures. In this study, we have established an easily applicable, reproducible, and cost-effective in vitro AV tissue culture model to study pathological mechanisms underlying CAVD. The valvular ECM and realistic VIC–VEC interactions mimic natural conditions more closely than VIC cultures or 3D environments. The application of various culture conditions enables the examination of different pathological mechanisms underlying CAVD and could lead to a better understanding of the molecular mechanisms that lead to VIC degeneration and AS. Our model provides a valuable tool to study the complex pathobiology of CAVD and can be used to identify potential therapeutic targets for slowing disease progression.

## 1. Introduction

Calcific aortic valve disease (CAVD), a major cause of aortic stenosis (AS), is the most frequent type of valvular disorder worldwide [1,2]. After an asymptomatic latent period of 10–20 years, untreated AS is associated with a poor prognosis, and the 1-year mortality rate nears 50% [3]. Due to a lack of medical treatment, aortic valve replacement, performed surgically (SAVR) or transcatheterally (TAVI) remains the gold standard of the treatment of symptomatic aortic valve stenosis [4,5].

CAVD is characterized by a complex multifactorial pathogenesis and was, for a long time, considered as a simple, passive process, but nowadays, CAVD is recognized as an actively regulated, cellularly driven, and slowly progressive disease [6,7,8,9,10]. Amongst the hallmarks of CAVD are the significant changes that occur in the composition, organization, and mechanical properties of the highly organized extracellular matrix (ECM) of the aortic valve (AV) [11,12]. The semilunar cusps are composed of three distinct layers, accurately described as the fibrosa, the spongiosa, and the ventricularis [9,13]. The cellular components of the aortic valve include a monolayer of valvular endothelial cells (VECs) on the outer surface of the leaflets and valvular interstitial cells (VICs), which populate each of the three layers [6,9]. VICs can differentiate into myofibroblast-like (aVIC) or osteoblast-like (obVIC) phenotypes and have been considered as the key players in the progression of CAVD [14,15]. Further, the composition and stiffness of the ECM may have a profound impact on the VIC phenotype [15,16]. VECs play an important role in maintaining valve homeostasis by regulating permeability, inflammatory cell adhesion, and paracrine signaling. However, they have also been indicated as key regulators in the initiation and early progression of CAVD via the recruitment of immune cells, dysregulation of protective nitric oxide (NO) signaling, or by undergoing endothelial-to-mesenchymal transition (EndMT) [17,18,19,20,21,22]. The role of VICs and VECs in the development and progression of CAVD is difficult to study, and models that can accurately replicate the pathological mechanism in CAVD are lacking [23]. Explanted calcified AVs from patients undergoing SAVR are of great value, but disease mechanisms cannot be extrapolated from the end-stage pathology [24]. In general, most research regarding CAVD is based on experiments using two-dimensional (2D) cell culture or artificially created three-dimensional (3D) environments of VICs, most commonly neglecting VECs. Simplistic 2D cell culture systems, created by the spontaneous aggregation of primary VICs or VECs from a high-density monolayer culture, contributed significantly to the better understanding of the pathobiology of CAVD but have many limitations, such as different environmental cues compared to natural tissues causing radical alterations in cell morphology and function [25,26]. Artificially created 3D systems provide symmetric adhesions and confinement more similar to the native ECM environment but usually consisting of a hydrogel matrix, which is unsuitable for studying the degenerative process over a longer period [15,27]. The co-culturing techniques also have some disadvantages, because maintenance of the quality and stability of the population of co-cultured species is a tedious task [28]. However, recently described novel 3D models with human VICs, which are suitable to investigate VIC phenotype changes as a result of both communication with valvular endothelial cells and exposure to pathological stimuli, provide promising tools to better understand the valve cell biology and pathological mechanisms underlying CAVD [29,30].

Nevertheless, animal studies are currently the only option to examine potential medical treatments to prevent the progression of CAVD over a longer period, but so far, there is no appropriate in vitro model available [23,31,32]. Hence, we developed a novel in vitro CAVD model with realistic VIC–VEC interactions that is more closely able to mimic natural conditions to study the cellular responses in degenerative processes of AVs, established varying culture conditions, and compared our findings to 2D cell cultures.

## 2. Material and Methods

### 2.1. Preparation of AV Leaflets and Application of In Vitro CAVD Model

**Figure 1:**
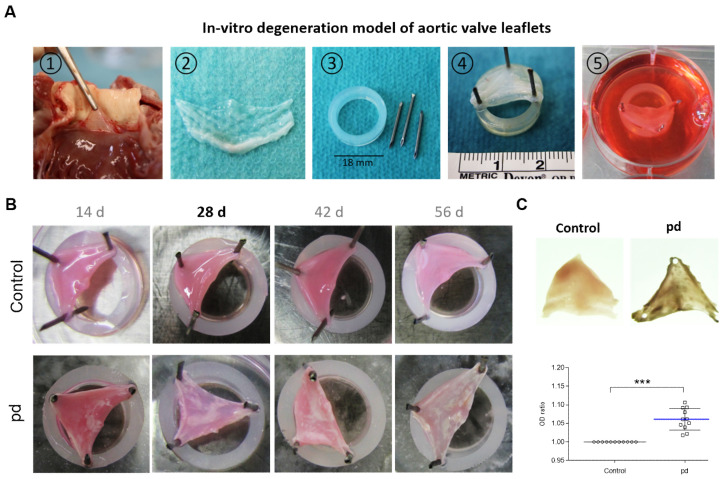
In vitro degeneration model of aortic valve leaflets. (**A**) Application of in vitro CAVD model: (1) Preparation of AV leaflets. (2) Excised AV leaflets after washing in PBS. (3) Required materials (silicon rubber rings and needles). (4) AV leaflet stretched on silicon rubber ring. (5) Cultivation of tensed AV leaflets. (**B**) Images of temporal progression of AV leaflet degeneration. White areas indicate calcified domains. (**C**) Representative transmitted light images of AV leaflets after 28 d cultivation and analysis of optical density (OD). Data (*n* = 8) are mean ± SEM. *p*-values are calculated by using Student’s t-test with Dunn’s multiple comparison post hoc test.; ***: *p* < 0.001. Pd, (pro-degenerative) condition.

Tricuspid aortic roots were excised from the hearts of healthy 6–9-month-old *Ovis aries* slaughtered in a local abattoir. Sheep were not killed specifically for the purpose of the present study, and no experiments were performed on living animals before slaughtering. Briefly, after removing the cardiac apex, the left heart was cut open alongside the lateral wall of the left ventricle through the mitral valve basis and the left auricular appendage. Afterward the aortic valve plane was opened by a straight cut along the commissure between the left ventricle and the aorta, preserving the leaflet anatomy. The AV leaflets were excised from the aortic root and washed multiple times in cold sterile phosphate-buffered saline (PBS, supplemented with 100 U/mL penicillin–streptomycin (P/S; Thermo Fisher Scientific, Waltham, MA, USA) and 1% amphotericin B (EurimPharm, Saaldorf-Surheim, Germany)) until blood residues were completely removed. For application of the CAVD model, AV leaflets were stretched with needles on silicon rubber rings with passive tension (Figure 1A). Care was taken that the AV leaflets were still slightly sagging and not so taut. For further details, see Figures S1 and S2.

### 2.2. Isolation and Culture of Primary Ovine VICs

Valvular interstitial cells (VICs) were isolated as described previously [33]. Briefly, excised leaflets were washed in cooled PBS, cut into small pieces, put into gelatine-coated (0.5%) cell culture flasks and cultured with Dulbecco’s modified Eagle’s medium (DMEM) containing 4.5 g/L glucose with GlutaMAX supplement (Invitrogen, Carlsbad, CA, USA) including 10% fetal calf serum (FCS; Sigma-Aldrich, St. Louis, MO, USA), 100 U/mL P/S, 1% non-essential amino acids (Sigma-Aldrich, St. Louis, MO, USA), and 1 μg/mL amphotericin B at 37 °C and 5% CO_2_ to allow the VICs to emigrate. Cells were used between passages 4 and 6.

### 2.3. In Vitro Degeneration

Valvular interstitial cells (VICs) and leaflets were cultured under pro-degenerative (pd) conditions (DMEM, 10% FCS, 10 mM β-glycerophosphate disodium salt hydrate (β-GP, Sigma-Aldrich, St. Louis, MO, USA), and 1.5 mM calcium chloride (CaCl_2_; Sigma-Aldrich, St. Louis, MO, USA)) or pro-calcifying (pc) conditions (DMEM, 5% FCS, and 2 mM sodium dihydrogen phosphate (Merck KGaA, Darmstadt, Germany)). For 2D VIC cultures, cells were seeded in 6- or 48-well plates and treated upon confluence for 7 days with medium change every 2–3 days. For immunohistochemical staining of cultured cells, VICs were seeded on gelatine-coated glass cover slips (10 mm in diameter; Glaswarenfabrik, Karl Hecht KG, Sondheim, Germany) placed in 6-well plates and cultured for 7 days using the aforementioned treatments. Leaflet cultures were placed in 6-well plates in 10 mL culture medium, and the cultivation period was expanded to 56 days with medium change once a week.

### 2.4. Optical Density Measurement

After cultivating the AV tissues according to different conditions and time durations, the leaflets were photo documented using a camera (PowerShot SX20 IS, Canon, Tokyo, JPN), a light pad (Slimlite LED, KAISER, Buchen, Germany), a self-made photo box, and a measuring stick (METRIC INCHES Devon^®^, Covidien, Dublin, Ireland). Optical-density measurements were accomplished using the image analysis software Image J 1.52a (National Institutes of Health (NIH)). After calibration of the scale, the leaflet outlines were encircled using polygon selections. Mean and median of the measurements inside the leaflets, and their area, were determined. The mean of the measurements points out the tissue intensity and comparing it to the measurements of the background yields the optical density of each leaflet.

### 2.5. Alizarin Red S Calcium Staining of 2D VIC Cultures

The degeneration level of VIC cultures was determined as described previously. Briefly, VIC monolayers were rinsed with PBS, fixed with neutral-buffered 4% formalin, and stained with alizarin red S (pH 4.2; Roth, Karlsruhe, Germany) solution. Observations and photographic records were made using an inverse microscope system (DM IL Type LED; Leica, Wetzlar, Germany) equipped with a digital camera (DFC425C) using LAS software version 3.8 (Leica DM IL Type LED, Wetzlar, Germany). For quantification, alizarin red S was extracted with 100 mM cetylpyridinium chloride monohydrate (CPC, Sigma-Aldrich, St. Louis, MO, USA). After 3 h of extraction (shaking, RT), absorbance was measured at 540 nm using a Tecan infinite M1000 pro microplate Reader (Tecan, Männerdorf, Switzerland).

### 2.6. Determination of Lactate Dehydrogenase, Alkaline Phosphatase, and Phosphate Levels in Supernatants

The levels of lactate dehydrogenase (LDH) in supernatants were measured by using the LDH Cytotoxicity Assay Kit according to manufacturer instructions (Thermo Fisher Scientific, Waltham, MA, USA). The assay relies on conversion of lactate to pyruvate via NAD+ reduction to NADH by LDH. Diaphorase then uses NADH to reduce a tetrazolium salt (INT) to a red formazan product that can be measured at 490 nm. The level of formazan formation is directly proportional to the amount of LDH released into the medium, which is indicative of cytotoxicity. Absorbance at 490 and 680 nm was measured using a Tecan infinite M1000 pro microplate Reader (Tecan, Männerdorf, Switzerland). Alkaline phosphatase (ALP) content was measured using the ALP colorimetric assay kit, according to manufacturer’s instructions (BioVision, Milpitas, CA, USA). The kit uses *p*-nitrophenyl phosphate (pNPP) as a phosphatase substrate, which turns yellow (405 nm) when dephosphorylated by ALP. Phosphate content was measured using the phosphate colorimetric assay kit, according to manufacturer’s instructions (BioVision, CA, USA). The assay utilizes a formulation of malachite green and ammonium molybdate, which forms a chromogenic complex with phosphate ions. The absorption of the latter complex was measured at 650 nm.

### 2.7. RNA Isolation and Semiquantitative Real-Time Polymerase Chain Reaction (qRT-PCR)

Total RNA from VIC cultures was isolated with a RNeasy mini kit (Qiagen, Hilden, Germany) according to the manufacturer’s instructions. AV leaflets were frozen in liquid nitrogen, crushed using a mortar and pestle, and lysed in TRIZOL (Thermo Fisher Scientific, Waltham, MA, USA) before purifying with a RNeasy mini kit. RNA was reverse transcribed using a commercial kit (Quantitect Reverse Transcription Kit, Qiagen, Hilden, Germany) and Biometra T3000 Thermocycler (Göttingen, Germany). Quantitative RT-PCR was performed using Promega SYBR Green PCR kit (Promega, Madison, WI, USA) on a real-time cycler (Applied Biosystems StepOnePlus; Thermo Fisher Scientific, Waltham, MA, USA). PCR protocol was as follows: starting with an initial step for 2 min at 50 °C, followed by 2 min at 95 °C. In all, 40 cycles were performed for 15 s at 95 °C and 30 s at 60 °C followed by single steps for 15 s at 95 °C, 1 min at 60 °C, and 15 s at 95 °C (primer sequences are shown in Table 1). The expression of the *RPL-29A* gene was used as a reference gene to normalize the results using the comparative 2^-ΔΔCt^ method.

### 2.8. SDS-PAGE and Western Blot Analysis

Analysis of smooth muscle alpha actin (α-SMA) and vimentin (VIM) was carried out for VIC cultures and AV leaflets. Cells were lysed directly on the plate with RIPA buffer (Sigma-Aldrich, St. Louis, MO, USA) containing PhosSTOP (Sigma-Aldrich, St. Louis, MO, USA) and Complete Mini protease inhibitor cocktail (Sigma-Aldrich, St. Louis, MO, USA) on ice, homogenized by pipetting up and down, and centrifuged at 14,000 rpm for 20 min at 4 °C. AV leaflet tissues were frozen in liquid nitrogen, crushed using a mortar and pestle, and lysed with RIPA buffer. Protein homogenates were separated on a 10% reducing SDS-polyacrylamide gel (Thermo Fisher Scientific, Waltham, MA, USA) using the Laemmli method and then transferred to nitrocellulose membranes (Bio-Rad, Berkeley, CA, USA). Detection of protein signals was performed with primary antibodies against vimentin (VIM),1:1000, cat. no.: GP53, Progen, Heidelberg, Germany) and alpha smooth muscle actin (α-SMA,1:1000, cat. no.: ab5694, Abcam, Cambridge, UK). For normalization, detection of housekeeper protein signals was performed on the respective nitrocellulose membranes with primary antibody against glyceraldehyde 3-phosphate dehydrogenase (GAPDH, 1:2000, cat. no.: C52118, Cell Signaling, Danvers, MA, USA). For detection of primary antibody signals, the following HRP-conjugated secondary antibodies were used: goat anti rabbit IgG (1:10000, cat. no.: 111-035-003, Dianova, Hamburg, Germany), goat-anti mouse IgG + IgM (1:10,000, cat. no.: 115-035-044, Jackson ImmunoResearch, West Grove, PA, USA), and goat anti guinea pig IgG (1:10,000, cat. no.: 106-035-003, Dianova). Molecular weight was determined using PageRuler Prestained Protein Ladder (cat. no.: 26616; Thermo Fisher Scientific, Waltham, MA, USA). Protein bands were visualized using Western Bright™ Quantum Western Blotting Detection System (Advansta, Menlo Park, CA, USA) following standard protocols. The membrane was digitalized using an Amersham Imager 600 (GE Healthcare, Freiburg, Germany) and analyzed for densitometry with ImageJ software 1.52a (National Institutes of Health, Bethesda, MD, USA).

### 2.9. Histological Staining

AV leaflets were washed in PBS, embedded in KP-CryoCompound (VWR Chemicals, Radnor, PA, USA), and cryopreserved with liquid nitrogen. Cryosections of 8 μm thicknesses were prepared (Leica CM1950 microtome, Wetzlar, Germany) and analyzed after staining with hematoxylin–eosin (HE), von Kossa, alizarin red S, and modified Movat’s pentachrome. For HE staining, sections were rinsed in distilled water and then incubated in hematoxylin solution (Thermo Fisher Scientific, Waltham, MA, USA), followed by differentiation in 5% acid alcohol. After washing under tap water, the sections were dehydrated through 70%, 80%, 95%, and 100% alcohol, and then stained with 2% *w/v* eosin b solution (Sigma Aldrich, Steinheim, Germany). For von Kossa staining, the sections were hydrated, incubated in 5% *w/v* silver nitrate solution (VWR Chemicals, Radnor, PA, USA), washed in 5% *w/v* sodium carbonate (Sigma Aldrich, Steinheim, Germany), and counterstained with nuclear fast red (Roth, Karlsruhe, Germany). For alizarin red S staining (Roth, Karlsruhe, Germany), sections were rinsed in distilled water and then stained with 2% *w/v* alizarin red S solution (pH 4.3), followed by dehydration though acetone, acetone–xylene (1:1), and xylene. For Movat’s pentachrome staining, the sections were hydrated and fixed in formalin, following Bouin’s solution (picric acid, acetic acid, and formaldehyde), and then sodium thiosulfate. After rinsing with distilled water, the sections were stained with Alcian blue, washed under tap water and stabilized with alkaline alcohol (3% ammonium hydroxide in ethanol). After intense washing under tap water, sections were stained with Verhoeff’s working solution (5% alcoholic hematoxylin, 10% ferric chloride, and Weigert’s iodine solution (potassium iodine and iodine)). After rinsing with distilled water, sections were stained with brilliant crocein acid fuchsin solution, washed with distilled water, stained with phosphotungstic acid solution (5%), washed with acetic acid (1%), and dehydrated in ethanol after another wash step. After staining in alcoholic saffron solution, sections were washed with distilled water, dehydrated with alcohol, and degreased with xylene. For AP staining, the sections were stained with nitrotetrazolium blue chloride (NBT)/5-brom-4-chlor-3-indoxylphosphat (BCIP) substrate solution (Thermo Fisher Scientific, MA, USA) for 30 min at 37 °C and then washed with distilled water. The stained sections were then sealed with Roti™ HistoKitt (Roth, Karlsruhe, Germany) and imaged under a Leica DM2000 microscope equipped with a digital camera (Leica DFC 425C, Wetzlar, Germany). Pictures of the alizarin red S- and AP-stained sections were quantified by digital image analyses with ImageJ software 1.52a. Clinical samples of calcified aortic valve tissue served as the positive control.

### 2.10. Immunohistochemistry

Sections and pre-washed cells on cover slips were fixed with formalin (4%) for 10 min and then incubated for 10 min in 0.25% Triton-X-100 in PBS, followed by three washing steps in PBS. After blocking with 5% BSA for 60 min, primary antibodies against von Willebrand factor (cat. no.: A0082, Dako, Agilent, CA, USA), VIM (cat. no.: GP53, Progen, Heidelberg, Germany), and α-SMA (cat. no.: ab5694, Abcam, Cambridge, UK) were incubated over night at 4 °C, followed by three washing steps with PBS. Then, sections or cells were incubated with secondary fluorescent antibodies (Alexa488 and Alexa546; Dianova, Hamburg, Germany) for 60 min and 4′,6-diamidino-2-phenylindole (DAPI; cat. no.: 6335, Carl Roth, Karlsruhe, Germany) for 10 min and were washed three times with PBS. After rinsing in distilled water, sections or cover slips were mounted on microscope slides. Immunofluorescent micrographs were taken using a DM2000 microscope, a DFC425C camera, and LAS software version 3.8 (Leica, Wetzlar, Germany).

### 2.11. Statistical Analysis

Statistical analysis was performed with Prism 6 software (GraphPad, San Diego, CA, USA) using Student’s *t*-test or nonparametric Kruskal–Wallis test with Dunn’s multiple comparison post hoc test. All data are reported as mean ± standard deviation (SD) or standard error of the mean (SEM). Significance levels were expressed as *p* < 0.05 (*), *p* < 0.01 (**), *p* < 0.001 (***).

## 3. Results

### 3.1. Degeneration of AV Leaflets Progresses over Time

**Figure 2:**
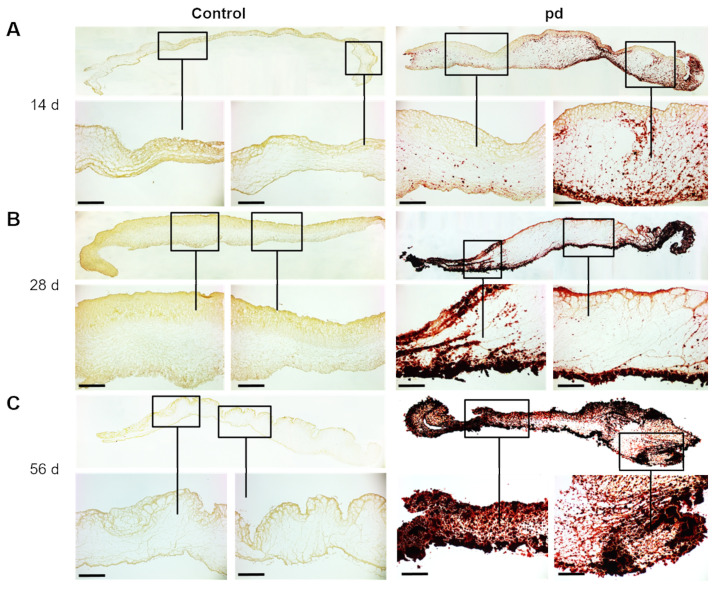
Temporal progression of AV leaflet degeneration. Alizarin red S staining of AV leaflets under pro-degenerative (pd) conditions (β-GP + CaCl_2_) after 14 d (**A**), 28 d (**B**), and 56 d (**C**) compared to control conditions. Red indicates sites of biomineralization. Scale bar indicates 100 µm. Representative images of five different experiments are shown.

Photo-optical images of AV leaflets point to a temporal progression of degeneration under pd (pro-degenerative) conditions (Figure 1B). After 14 d of cultivation, the first calcified domains were visible, but the shape of the AV leaflets was only slightly impaired at the commissures. With increasing culture duration under pd conditions, both the calcified domains continued to increase, and the shape of the AV leaflets was clearly altered, particularly at the commissures compared to control conditions. In experiments aiming at long-term cultivation, a number of cultures had to be terminated due to contamination and were replaced. There was no evident increase in contamination rate associated with a certain treatment modality. Further, after a 56 d cultivation period, the formation of calcium nodules was clearly visible, and the AV leaflets exhibited a heightened stiffness. The significantly increased OD after 28 d of cultivation under pd conditions compared to control conditions confirmed the strong degeneration of AV leaflets in our CAVD model (*p* < 0.001, Figure 1C). Alizarin red S staining displayed that degeneration begins in the outer layers, primarily in the ventricularis layer of the AV leaflet, before spreading into the spongiosa layer (Figure 2A). After 28 d of cultivation, distinct calcium accumulation could be detected in both the ventricularis and the fibrosa layer (Figure 2B). Ultimately, after 56 d of cultivation, massive calcium accumulation could be substantiated in all three layers of the AV leaflets (Figure 2C). Additional von Kossa staining confirmed our findings (Figure S3). However, if the AV leaflets were not stretched on silicon rubbers, they convolved during cultivation, leading to a more progressive and stronger degeneration and loss of the layer-dependent progression (Figure S4).

### 3.2. Progressing Degeneration Leads to ECM Disruption

**Figure 3:**
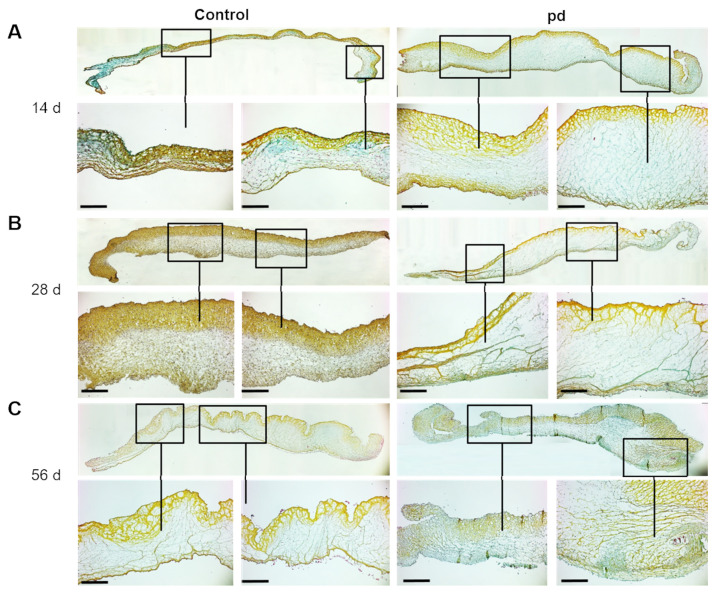
Temporal progression of ECM remodeling of AV leaflets. Movat’s pentachrome staining of AV leaflets under pro-degenerative (pd) conditions (β-GP + CaCl_2_) after 14 d (**A**), 28 d (**B**), and 56 d (**C**) compared to control conditions. Scale bar indicates 100 µm. Representative images of five different experiments are shown.

Movat’s pentachrome stain was used to examine alterations of the ECM architecture. After 14 d of cultivation under control conditions, the AV leaflets showed a defined trilaminar ECM architecture and uniform thickness (Figure 3A). Under pd conditions, the AV leaflets were distinctly thickened, particularly based on extensive condensation of the spongiosa layer. After 28 d, the thickening of the fibrosa layer under control conditions is based on a qualitatively increased VIC density due to high proliferation rates (Figure 3B and Figure S5). Under pd conditions, a distinct structural disorganization could be detected in the layered ECM structure. The amount of proteoglycans (blue) was increased in the spongiosa layer and the collagen- (yellow) rich fibrosa layer was clearly disrupted. After 56 d of cultivation, the trilaminar organization of AV leaflets was still discernible (Figure 3C). The AV leaflets were unevenly thickened, and the ECM structure was clearly disrupted under pd conditions. One of the most notable changes in the ECM was the enrichment of proteoglycans throughout in all layers. Additional von Kossa and H&E staining substantiated our findings (Figure S6).

### 3.3. The Endothelial Cell Layer of AV Leaflets Is Disrupted under PD Conditions

**Figure 4:**
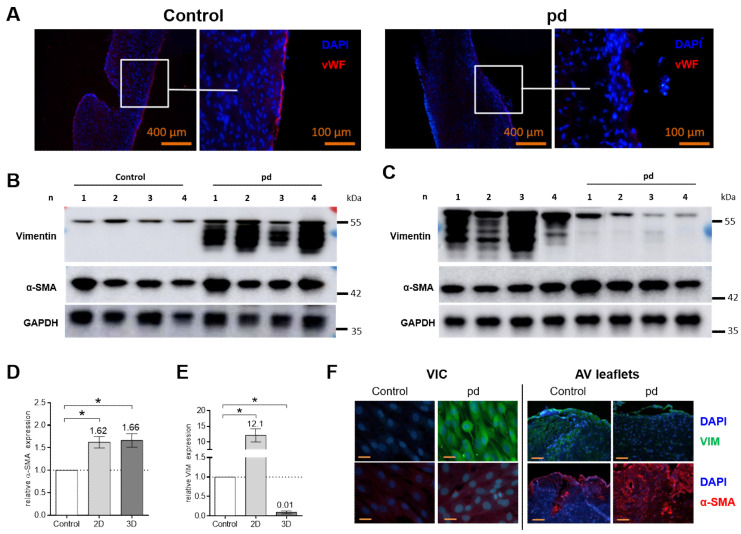
Analysis of endothelial layer and expression of VIM and α-SMA. (**A**) Immunohistological images with antibodies against von Willebrand factor (vWF) of AV leaflets under pro-degenerative (pd, β-GP + CaCl_2_) and control conditions after a 28 d cultivation period. Representative images of four different experiments are shown. Scale bar indicates 400 or 100 µm. Western blot images of 2D VIC cultures after 7 d (**B**) and AV leaflets after 28 d (**C**) for vimentin (VIM), smooth muscle alpha actin (α-SMA) and glyceraldehyde 3-phosphate dehydrogenase (GAPDH) under pd and control conditions. Density analysis for quantification of α-SMA (**D**) and VIM (**E**) in 2D VIC cultures and AV leaflets. Data (*n* = 4) are mean ± SEM. *p*-values are calculated by using Kruskal–Wallis test with Dunn’s multiple comparison post hoc test. *: *p* < 0.05. Data were normalized to GAPDH and expressed relative to control conditions. (**F**) Immunohistological images with antibodies against VIM (green) and α-SMA (red) of 2D VIC cultures and AV leaflets under pd and control conditions. Representative images of four different experiments are shown. DAPI, 4′,6-diamidino-2-phenylindole; VIC, Valvular interstitial cells.

Under pd conditions, lactate dehydrogenase (LDH) levels were not higher as compared to control conditions up to a 28 d cultivation period (Figure S7). However, under control conditions, immunohistological staining of von Willebrand factor (vWF) after a 28 d cultivation period displayed a continuous layer of attached endothelial cells, while in contrast, cultivation under pd conditions clearly led to a disruption and ablation of the endothelial cell layer of the AV leaflets (Figure 4A and Figure S8).

### 3.4. Protein Expression

Based on our histological findings, we selected a 28 d cultivation period for protein analysis of AV leaflet cultures and compared our finding with VIC cultures (7 d). The expression of alpha smooth muscle actin (α-SMA) was increased under pd conditions both in VIC cultures and in AV leaflets compared to control conditions (*p* < 0.05 Figure 4B–D). In contrast, the expression of vimentin (VIM) increased significantly under pd conditions in VIC cultures (*p* < 0.05) and decreased in AV leaflet cultures (*p* < 0.05) compared to that in the respective control conditions (Figure 4E). Immunohistochemical staining confirmed the findings of Western blot analyses (Figure 4F).

### 3.5. Gene Expression Is Altered in AV Leaflets Compared to VIC Cultures under PD Conditions

**Figure 5:**
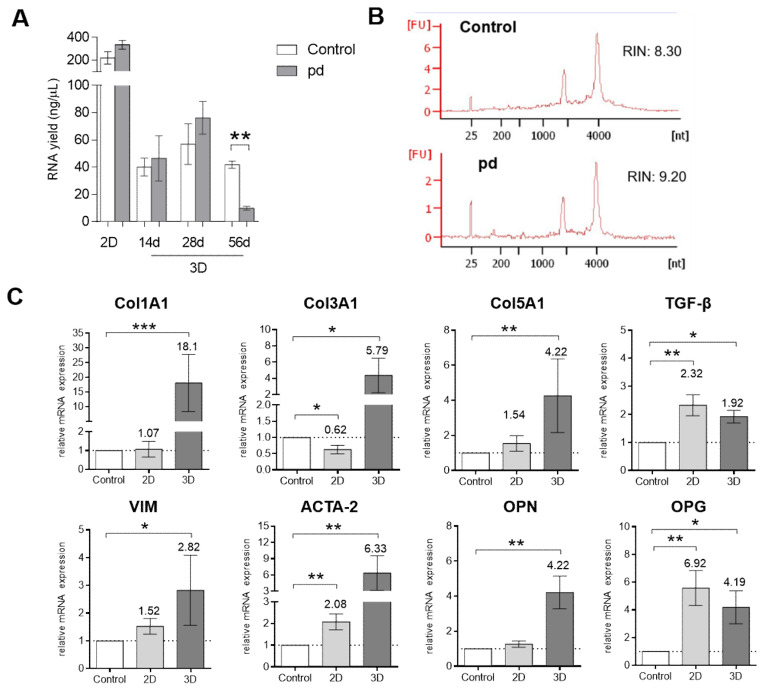
Gene expression analysis. (**A**) Comparative RNA yields of 2D VIC cultures (7 d) and AV leaflets after 14, 28, and 56 d cultivation under pro-degenerative (pd) conditions (β-GP + CaCl_2_, dark column) compared to control conditions (white column). (**B**) Representative RNA integrity numbers (RINs) of AV leaflets after 28 d cultivation. (**C**) Analysis of gene expression of 2D VIC cultures (gray column, 7 d) and AV leaflets (dark column, 28 d) under pd conditions for alpha-1 type I collagen (Col1A1), alpha-1 type III collagen (Col3A1), alpha-1 type V collagen (Col5A1), transforming growth factor beta 1 (TGF-β1), vimentin (VIM), alpha smooth muscle actin (ACTA2), osteopontin (OPN), and osteoprotergerin (OPG) compared to control conditions (white column). Data (*n* = 6–8) are mean ± SEM. *p*-values are calculated by using Kruskal–Wallis test with Dunn’s multiple comparison post hoc test. *: *p* < 0.05; **: *p* < 0.01; ***: *p* < 0.001. FU, fluorescence units.

In AV leaflets, the RNA yield was at the same level after 14 and 28 d cultivation periods under control and pd conditions (Figure 5A). Unfortunately, due to far advanced degeneration, the RNA yield of AV leaflets cultured under pd conditions was very low after the 56 d cultivation period. Therefore, gene expression analysis was implemented with 14 and 28 d cultures and compared to VIC cultures. Analysis of the RNA integrity number (RIN) in AV leaflets after the 28 d cultivation period showed good quality of the isolated RNA both under control conditions (8.30) and under pd conditions (9.20, Figure 5B). We found that gene expression of alpha-1 type I collagen (Col1A1), alpha-1 type III collagen (Col3A1), and alpha-1 type V collagen (Col151) was significantly upregulated in AV leaflets under pd conditions after 28 d compared to control cultures (Col1A1: 18.1 fold, *p* < 0.001; Col3A1: 5.79 fold, *p* < 0.05; Col5A1: 4.22 fold, *p* < 0.01; Figure 5C). In VIC cultures, expression of Col3A1 was downregulated (0.62 fold, *p* < 0.05) under pd conditions, while Col1A1 and Col5A1 showed no significant changes. Moreover, the expression of collagens was not increased after the 14 d cultivation period (Figure S9). Expression of transforming growth factor beta 1 (TGF-β), alpha smooth muscle actin (ACTA2), and osteoprotergerin (OPG) was significantly upregulated both in VICs cultures (TGF-β: 2.32 fold, *p* < 0.01; ACTA-2: 2.08 fold, *p* < 0.01; OPG: 6.92 fold, *p* < 0.01) and AV leaflets (TGF-β: 1.92 fold, *p* < 0.05; ACTA-2: 5.33 fold, *p* < 0.01; OPG: 4.19 fold, *p* < 0.05) under pd conditions compared to the respective control cultures. In contrast, vimentin (VIM) and osteopontin (OPN) were significantly upregulated under pd conditions in AV leaflets (VIM: 1.92 fold, *p* < 0.05; OPN: 4.22 fold, *p* < 0.01).

### 3.6. Inorganic-Phosphate-Induced, AP-Independent Degeneration

**Figure 6:**
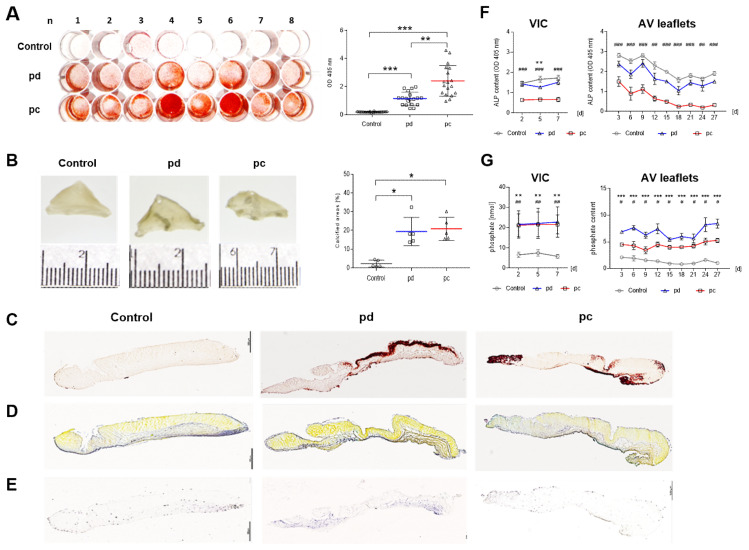
Comparison of pro-degenerative and pro-calcifying conditions. (**A**) Alizarin red S staining and quantification of 2D VIC cultures (*n* = 16) after 7 d under pro-degenerative (pd, β-GP + CaCl2; blue line) and pro-calcifying (pc, NaH_2_PO_4_, red line) conditions compared to control conditions. (**B**) Images and transmitted light images of AV leaflets after 28 d cultivation under pd and pc conditions and analysis of optical density (OD). Data (*n* = 6) are mean ± SEM. Alizarin red S (**C**), Movat’s pentachrome (**D**), and AP staining (**E**, purple areas) of AV leaflets under pd, pc, and control conditions after 28 d cultivation. Scale bar indicates 100 µm. Representative images of five different experiments are shown. Analysis of AP (**F**) and phosphate (**G**) in supernatants of 2D VIC cultures (d2–d7) and AV leaflet cultures (d3–d27) under pd (blue line) and pc (red line) conditions compared to control conditions (gray line). Data (*n* = 5) are mean ± SEM. *p*-values (*: pd vs. control, #: pc vs. control) are calculated by using Kruskal–Wallis test with Dunn’s multiple comparison post hoc test. * and #: *p* < 0.05; ** and ##: *p* < 0.01; *** and ###: *p* < 0.001.

Because degeneration of VICs is often induced by treatment with inorganic phosphate, we cultured AV leaflets under pro-calcifying (pc) conditions and compared the findings with our established pd conditions. The degeneration of VIC cultures was distinctly accelerated under pc conditions compared to pd conditions as demonstrated by alizarin red S staining (*p* < 0.01, Figure 6A and Figure S10). In AV leaflets, both conditions increased the calcified areas (*p* < 0.05, Figure 6B) and calcium accumulation (Figure 6C). Further, a distinct structural disorganization could be detected in the layered ECM structure under both conditions (Figure 6D). Additional von Kossa and H&E staining substantiated our histological findings (Figure S11). Expression of AP was remarkably decreased under pc conditions both in AV tissue (Figure 6E) and in supernatants of VIC (*p* < 0.001) an AV leaflet cultures (*p* < 0.01) at all points compared to control conditions (Figure 6F). Further, phosphate content in supernatants was significantly higher under pd and pc conditions compared to that in control conditions both in VIC cultures (pd and pc, *p* < 0.001) and AV leaflets (pd, *p* < 0.001; pc, *p* < 0.05) during the entire cultivation period (Figure 6G).

## 4. Discussion

Even though our understanding of cellular and molecular mechanisms in the progression of CAVD has increased enormously in the last years, the lack of reproducible tissue models mimicking natural conditions and accurately replicating pathological mechanisms has proven to be challenging for researchers in this field [23,24,31,34,35]. The maladaptations of the highly organized valvular ECM, which is constantly remodeled, either enzymatically or non-enzymatically are not simply a consequence of impaired valve cells but rather contribute to the progression of CAVD by altering various fundamental biological processes [11,36,37,38,39]. In this study, we utilized AV tissue culture as a novel in vitro CAVD model, deployed different pro-degenerative conditions, and conducted a comparative analysis of simplistic 2D VIC cultures. The degeneration of AV leaflets progressed over time, commencing with the first visible calcified domains after 14 d and winding up in the distinct formation of calcium nodules, heightened stiffness, and clear disruption of the ECM after 56 d. Both the expression of pro-degenerative genes and the myofibroblastic differentiation of VICs were altered in AV leaflets compared to those in VIC cultures. The applied in vitro tissue culture model of AV leaflets provides a valuable tool to study complex pathological processes of CAVD and can be used to identify potential therapeutic targets. Moreover, by varying culture conditions (osteogenic or phosphate-mediated), the examination of different aspects of CAVD becomes feasible.

In general, 2D cell culture systems are indispensable tools and of great value to study pathological mechanisms in CAVD [14,40]. Unfortunately, they do have some appertaining flaws. Because most 2D cell cultures models are adhesion dependent, the cells are cultured on flat, coated polystyrene plastic dishes and exhibit a different structural and functional behavior compared to natural environments [26,41,42]. The unpredictability of 2D cultures, due to the lack of cell–cell and cell–extracellular environment interactions, which are responsible for cell differentiation, proliferation, vitality, expression of genes and proteins, responsiveness to stimuli, drug metabolism, and other cellular functions, increases the cost and failure rate of new drug discovery and clinical trials [26,42]. Another drawback of 2D monolayer culture is that cells have unlimited access to medium ingredients such as oxygen, nutrients, metabolites, or signal molecules and issues caused by the growth media and expansion of cells can result in toxic waste products, dead cells, nutrition depletion, and damage of the environment the cells are in [26,42]. Despite these disadvantages, 2D cell cultures are still used for the majority of cell cultures, because they are less expensive than some other systems, well established, and typically easier to analyze. Further, there is a lot of literature, to which current results and outcome measures can be compared [26,41,42]. Artificially created 3D environments of VICs are more physiologically relevant and predictive than 2D cultures and exhibit a higher degree of structural complexity and homeostasis but are time consuming, labor intensive, and expensive [27,33,41,42]. Furthermore, 3D cultures created from specific tissues (e.g., basement membrane extracts) can contain undesirable components such as viruses or growth factors. It also must be mentioned that the microscopical analysis of 3D cultures is accompanied by technical challenges, while 2D cultures can be analyzed by almost any kind of imaging [27,33,41,42].

Our in vitro AV leaflet tissue culture model is easily applicable, reproducible, and cost effective and could be a major alternative to animal testing. The complex pathological processes underlying CAVD can be examined in a natural environment with a native valvular ECM and realistic VIC–VEC interactions. Our model can be used to identify potential drug targets for slowing disease progression or even reversing and curing it and may accelerate the discovery and validation of a drug-based therapy for CAVD.

CAVD is an actively regulated, slowly progressive disease with a long asymptomatic latent period usually of 10–20 years [1,2,6]. In 2D VIC cultures, the degeneration progresses fairly quickly, with an obvious calcium accumulation already after 7 d, while in tissue culture, the degeneration of AV leaflets is distinctly slower. Calcified domains are initially visible after 14 d and progress slowly over time up to 56 d. So far, no in vitro CAVD model has proven suitable for long cultivation periods. Three-dimensional environments usually consist of a hydrogel matrix. In the degeneration process, cellular signals promote qVICs to become activated (aVICs), which results in an increased secretion of matrix metalloproteinase (MMP) and leads to an enhanced degradation of the ECM. Consequently, 3D cell cultures of VICs on a hydrogel basis dissolve with progressing degeneration, and this makes them unsuitable for prospective long-term studies. With our tissue culture model, the cultivation period of AV leaflets for 4 to 8 weeks is unproblematic, future-oriented, and could also be expanded up to 3 months or even longer.

It was recently demonstrated that media culture conditions impact the calcification potential of primary human aortic VICs [43]. In this study, we applied two different culture conditions. Pro-degenerative media induces AP-dependent degeneration due to the organic phosphate source being β-GP [43,44,45,46]. Under pc conditions, containing inorganic phosphate, calcification is induced independent of AP activity by high phosphate availability [43,47,48,49]. While in 2D VIC cultures, the degeneration is distinctly accelerated under pc conditions compared to that in pd conditions, in AV tissue culture, the calcium accumulation is increased similarly. However, it is still obscure whether different culture conditions model divergent pathologies of CAVD. While supplementation with β-GP is generally used to induce calcification by promoting osteogenic differentiation, the elevated phosphate uptake is presumably the mediator of VIC degeneration in pc media [43,44,47]. Application of various culture conditions enables the examination of different pathological mechanisms underlying CAVD and could lead to a better understanding of the molecular processes that lead to VIC degeneration and AS.

CAVD is also characterized by alterations of the valvular ECM architecture, which exerts an important role in mediating pro-degenerative events [11,12,36]. The changes in ECM that occur in the organization and composition during the progression of CAVD deteriorate the mechanical properties of the valve and ultimately result in stiffened stenotic leaflets that obstruct flow and compromise cardiac function [6,11,12,36,39]. In our AV tissue culture model, progressing degeneration results in clear disruption and distinct structural disorganization of the ECM. While calcium accumulation begins in the outer layers of the AV leaflets, mainly in the ventricularis layer before spreading into the spongiosa layer, the thickening of the leaflets is primarily based on extensive condensation of the spongiosa layer. Despite massive calcium accumulation in all three layers after 56 d, the trilaminar organization of the AV leaflets was still discernible. In contrast to usually applied 3D cell culture systems, with our AV tissue culture model, it is possible to study ECM alterations during the progression of CAVD and to conduct layer-specific analyses.

Studies have shown dramatic morphological and biochemical differences between cells grown on 2D plastic substrates and in a 3D environment [25,26,41,42,50]. Comparing 2D VIC cultures and AV tissue culture, our data support the influence of the ECM on myofibroblastic differentiation and gene expression. The differentiation of VICs into myofibroblast-like (aVICs) phenotypes plays a crucial role in maintaining valve homeostasis and integrity and is considered a key mechanism in the progression of CAVD [14,15]. In our study, α-SMA was consistently upregulated in VIC cultures and AV tissue cultures, while VIM increased in VIC cultures but decreased in tissue culture. In AV tissue, VICs are embedded in a realistic ECM, consequently the secretion of ECM components was not increased as in VICs cultures. Further, higher expression of collagens and OPN was only detectable in AV tissue cultures. The variations potentially caused by the composition and stiffness of the valvular ECM underline its role in the progression of CAVD. In addition to the profound impact on VIC phenotypes, the valvular ECM also triggers expression of essential ECM components for further remodeling processes.

Endothelium damage is an early feature of CAVD and favors the accumulation of calcium and lipids, the infiltration of inflammatory cells, and the expression of pro-calcifying factors in the progression of CAVD [2,51,52]. In our model, the endothelial cell layer of AV leaflets was clearly impaired and disrupted under pd conditions compared to control conditions. Consequently, AV tissue culture enables researchers both to examine the role of the endothelial layer in the pathogenesis of CAVD and to explore potential approaches to prevent endothelial dysfunction. However, for further studies of endothelial function in our CAVD model, advanced investigations regarding the viability of VECs under both control and pd conditions are necessary.

Further, our model provides realistic VIC–VEC interactions. In general, 2D cell cultures are usually monocultures and allow the study of only one cell type. Indeed, co-culturing systems enable the analysis of interactions between cell populations but commonly lack a natural ECM [28,53]. However, VIC–VEC interactions play an important role in the pathogenesis of CAVD [14,15,22,54]. For instance, the potential of VECs undergoing endothelial-to-mesenchymal transition (EndMT) is a potential trigger and contributor to CAVD [22,55,56]. VECs can acquire a mesenchymal phenotype wherein the expression of endothelial markers and endothelial functional capacity is lost, but the expression of mesenchymal cell markers, such as α-SMA is upregulated [20,57]. In the aortic valve, EndMT can be induced by altered ECM but also inhibited by cross-talk interactions with VICs [22,56]. Further, dysregulation of protective nitric oxide (NO) signaling as well as the recruitment of immune cells by VECs may also be prevented by crosstalk with VICs [18,19].

However, the applied AV tissue culture model also has some limitations. One limitation is that the AV leaflets are cultured under passive tension. Hemodynamic forces (such as hypertension, elevated stretch, or shear stresses) experienced by the valve leaflets can cause tissue remodeling and inflammation, which may lead to calcification, stenosis, and ultimate valve failure. Another one is the missing direct contact with blood cells and factors of the circulatory system, which may also be a major contributor to altered VEC plasticity. Prospective, future improvements should focus on the application of dynamic shear stress and realistic mechanical forces as well as the adjustment of culture conditions to include cells and factors of the bloodstream.

## 5. Conclusions

In this study, we have established an easily applicable, reproducible, and cost-effective in vitro AV tissue culture model to study the pathological mechanisms underlying CAVD. The valvular ECM and realistic VIC–VEC interactions mimic natural conditions more closely than VIC cultures or artificially created 3D environments. Application of various culture conditions enables the examination of different pathological mechanisms underlying CAVD and could lead to a better understanding of the molecular mechanisms that lead to VIC degeneration and AS. Our model provides a valuable tool to study the complex pathobiology of CAVD and can be used to identify potential therapeutic targets for slowing disease progression.

## Figures and Tables

**Table 1:** Primer sequences.

Gene	Forward Sequences (5′–3′)	Reverse Sequences (5′–3′)
*RPL-29A*	CCAAGTCCAAGAACCACACC	TATCGTTGTGATCGGGGTTT
*ACTA2*	TAGAACACGGCATCATCACC	TGAGAAGGGTTGGATGCTCT
*COL1A1*	AAGACATCCCACCAGTCACC	TAAGTTCGTCGCAGATCACG
*COL3A1*	GACATAGAGGCTTTGATGGACGA	CACTTCCTCGAGCTCCATCG
*COL5A1*	CGAGAACCCGGATGAGAACC	GGCCTCCGATCCCTTCATAGA
*VIM*	GACCTGGAGCGTAAAGTGGA	CTCTTGAATCTGGGCCTGAA
*TGF-β*	GAGCCAGAGGCGGACTACTA	TCGGACGTGTTGAAGAACAT
*OPN*	GATGGCCGAGGTGATAGTGT	TCGTCTTCTTAGGTGCGTCA
*OPG*	GCGTGTGTGAATGTGAGGAG	CGAGAAGAACCCATCTGGAC

## Data Availability

The data that support the findings of this study are available from the corresponding authors upon reasonable request.

## Supplementary Materials

The following are available online at https://www.mdpi.com/article/10.3390/biomedicines9050474/s1, Figure S1: Preparation of AV leaflets from ovine hearts. Figure S2: Application of in vitro CAVD model. Figure S3: Temporal progression of AV leaflet degeneration. Figure S4: Comparison of AV leaflets cultured under pro-degenerative conditions with and without tension. Figure S5: Density of VICs in fibrosa layer. Figure S6: Temporal progression of ECM remodeling of AV leaflets. Figure S7: Analysis of LDH secretion. Figure S8: Analysis of endothelial layer. Figure S9: Gene expression analysis of AV leaflets after 14 d. Figure S10: Comparison of pro-degenerative and pro-calcifying conditions. Figure S11: Temporal progression of AV leaflet degeneration.
