# Supplementary material for: Reproducible In Vitro Tissue Culture Model to Study Basic Mechanisms of Calcific Aortic Valve Disease: Comparative Analysis to Valvular Interstitials Cells
Source: Biomedicines. 2021 Apr 26;9(5):474. doi: 10.3390/biomedicines9050474 (PMC8146785; doi:10.3390/biomedicines9050474)
Supplement: Supplementary file 1 [file biomedicines-09-00474-s001.zip › biomedicines-1139420-supplementary.pdf]

biomedicines

MDPI

Supplementary Materials

Reproducible In Vitro Tissue Culture Model to Study Basic Mechanisms of Calcific Aortic Valve Disease: Comparative Analysis to Valvular Interstitials Cells

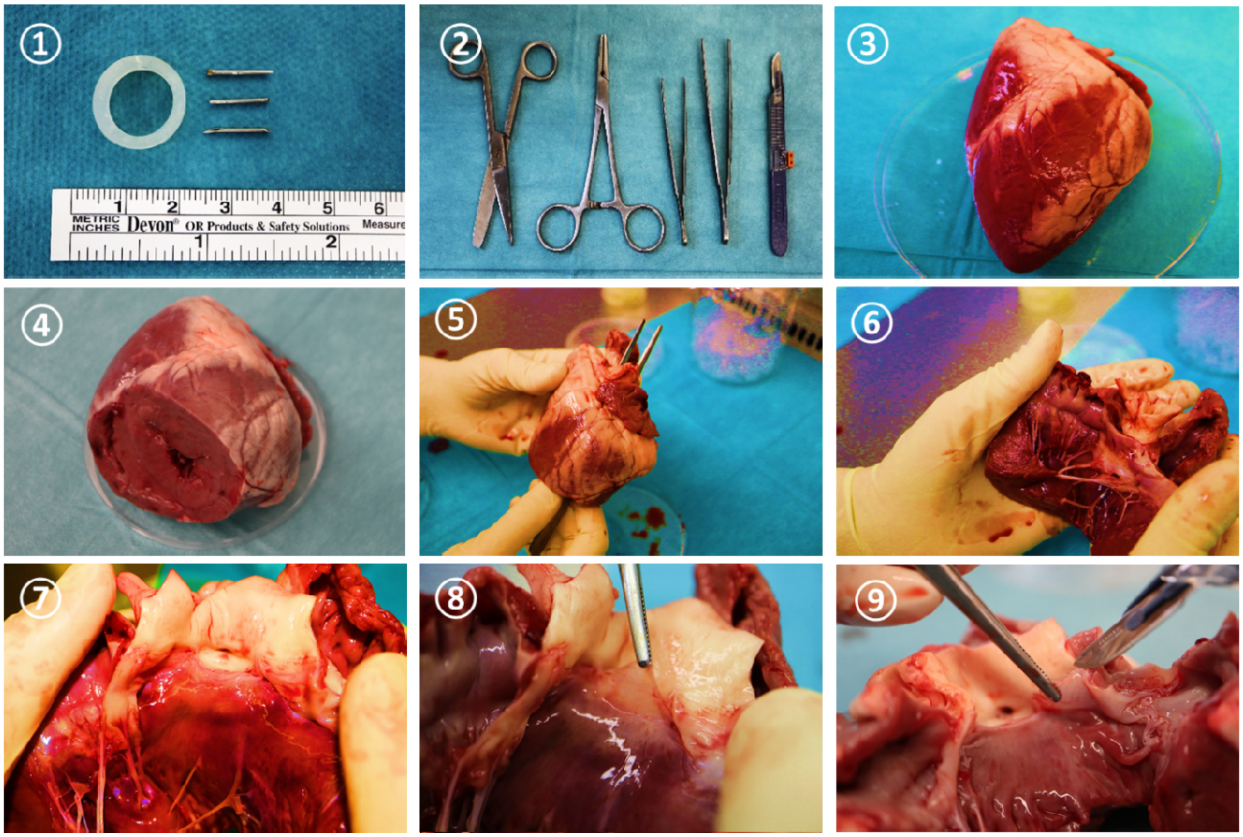

Figure S1. Preparation of AV leaflets from ovine hearts. (**1**) Required materials (silicon rubber rings and needles). (**2**) Instruments for preparation (ltr): scissor, needle holder, delicate and rough pincette and scalpel. (**3**) Whole ovine heart after removal of excessive tissue and pericard. (**4**) Ovine heart after removing the cardiac apex. (**5**) Orientation with a rough pincette through the opened left ventricle to the aorta. (**6**) Opened left ventricle with the view to the mitral valve. (**7**) View to the opened aorta and the three leaflets of the aortic valve. (**8**) Stretched AV leaflet using a pincette. (**9**) Excising a leaflet of the aortic valve by using scalpel and pincette.

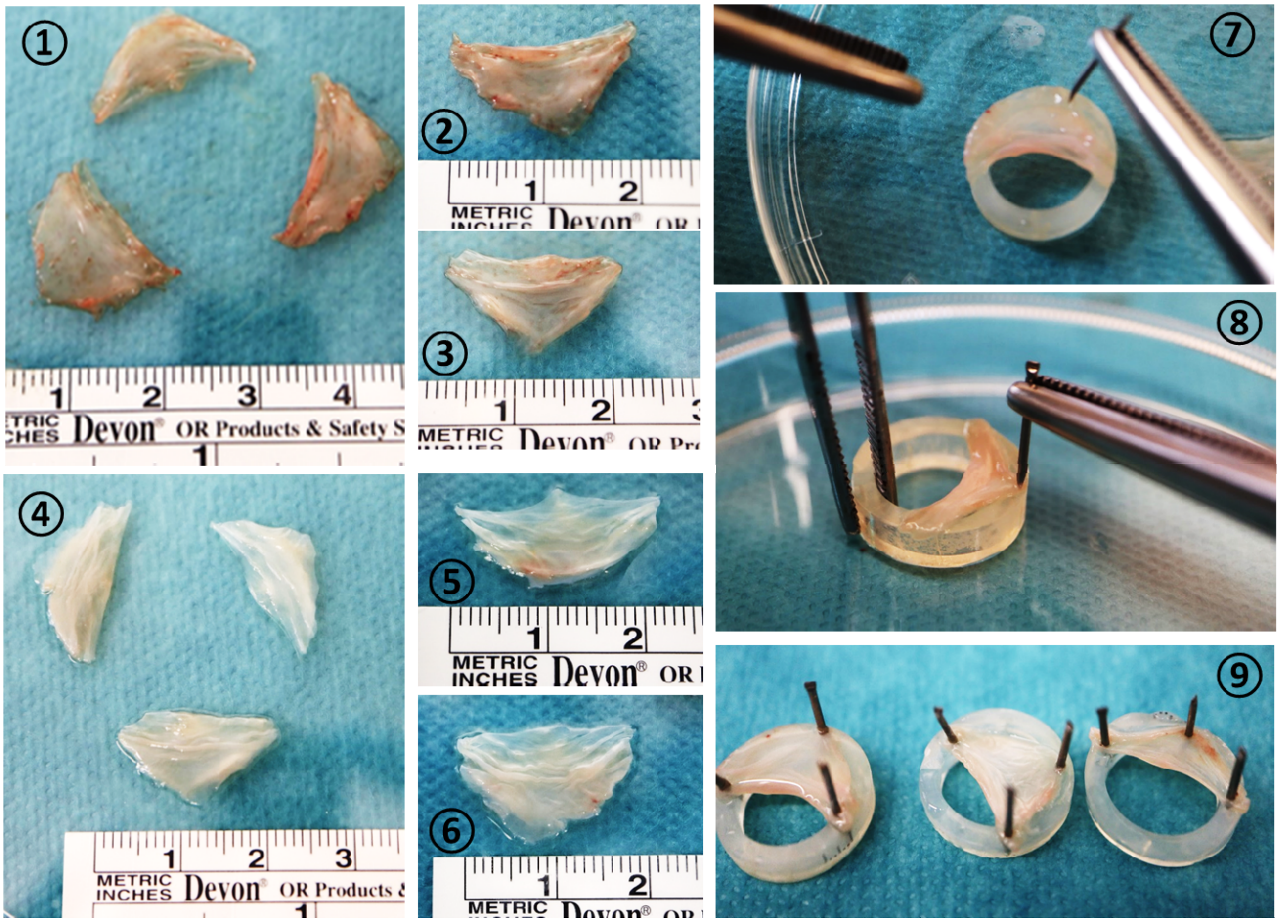

Figure S2. Application of *in-vitro* CAVD model. (**1**–**3**) Ovine aortic valve leaflets immediately after excising. (**4**–**6**) AV leaflets after washing multiple times with cold sterile PBS (supplemented with 2 % P/S and 1 % Amp B). (**7**) Stretching the AV leaflet on a rubber ring. (**8**) Attaching the leaflet on the rubber ring with a three needles using a needle holder and a pincette. (**9**) Stretched AV leaflets with passive tension.

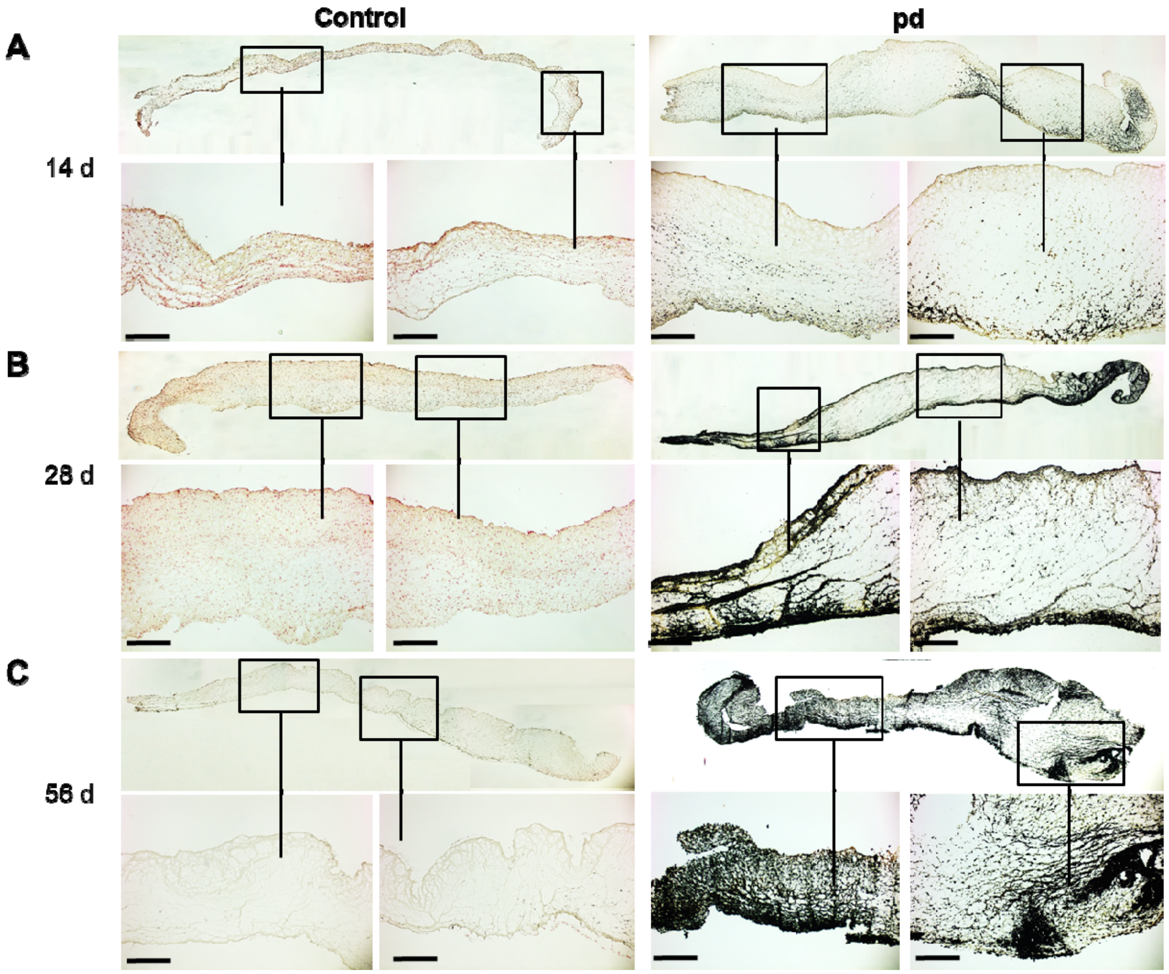

Figure S3. Temporal progression of AV leaflet degeneration. Von Kossa staining of AV leaflets under pro-degenerative (pd) conditions (β-GP + $CaCl_2$) after 14 d (**A**), 28 d (**B**) and 56 d (**C**) compared to control conditions. Black colour indicates sites of biomineralization. Scale bar indicates 100 µm. Representative images of five different experiments are shown.

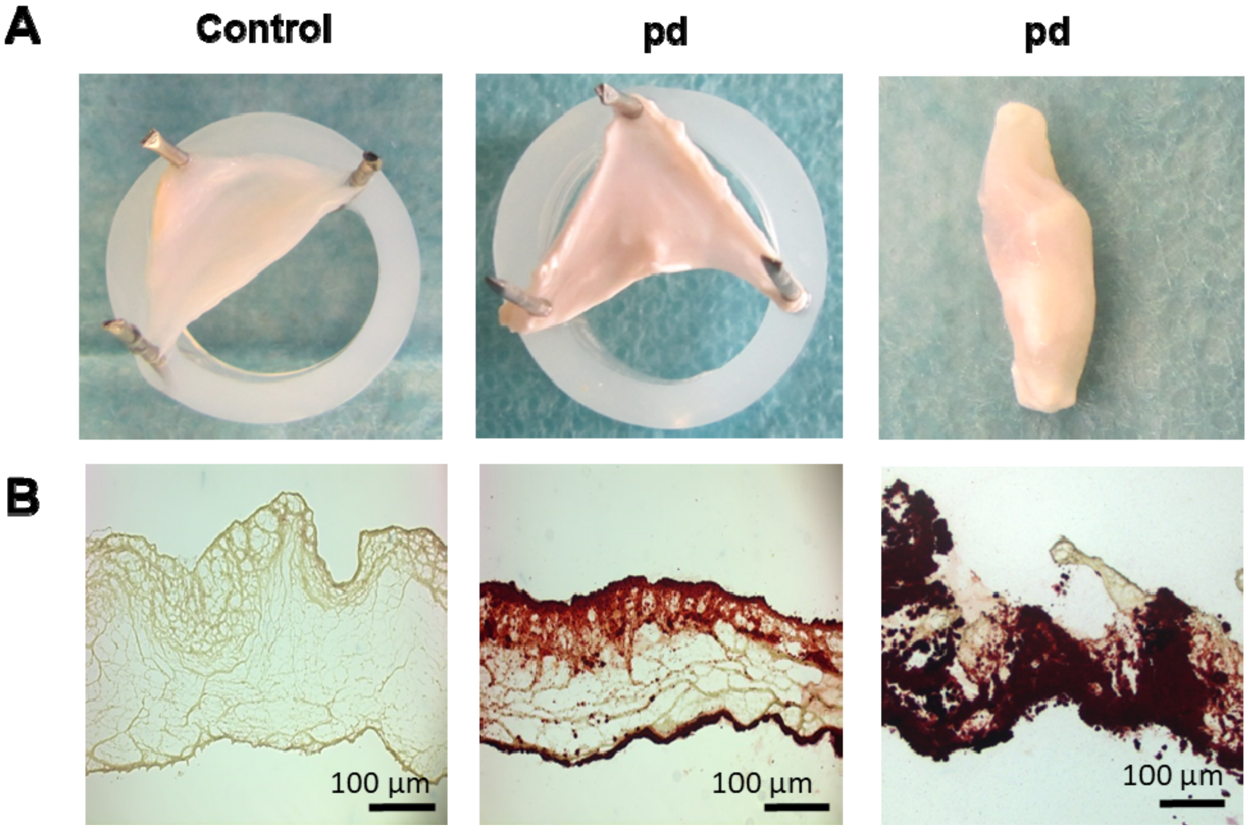

Figure S4. Comparison of AV leaflets cultured under pro-degenerative conditions with and without tension. Images of AV leaflets (**A**) and representative alizarin red S staining (**B**) after 28 d cultivation under pro-degenerative (pd) conditions. Scale bar indicates 100 µm.

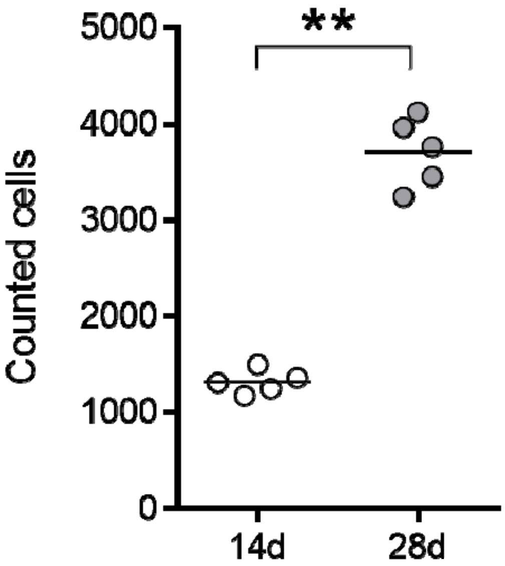

Figure S5. Density of VICs in fibrosa layer. Quantitative analysis of nucleuses of haematoxylin-eosin (HE) stained AV-tissue after 14 d and 28 d cultivation under control conditions ($n = 5$). p-values are calculated by using Student's t-test with Dunn's multiple comparison post hoc test. ** : $p < 0.05$.

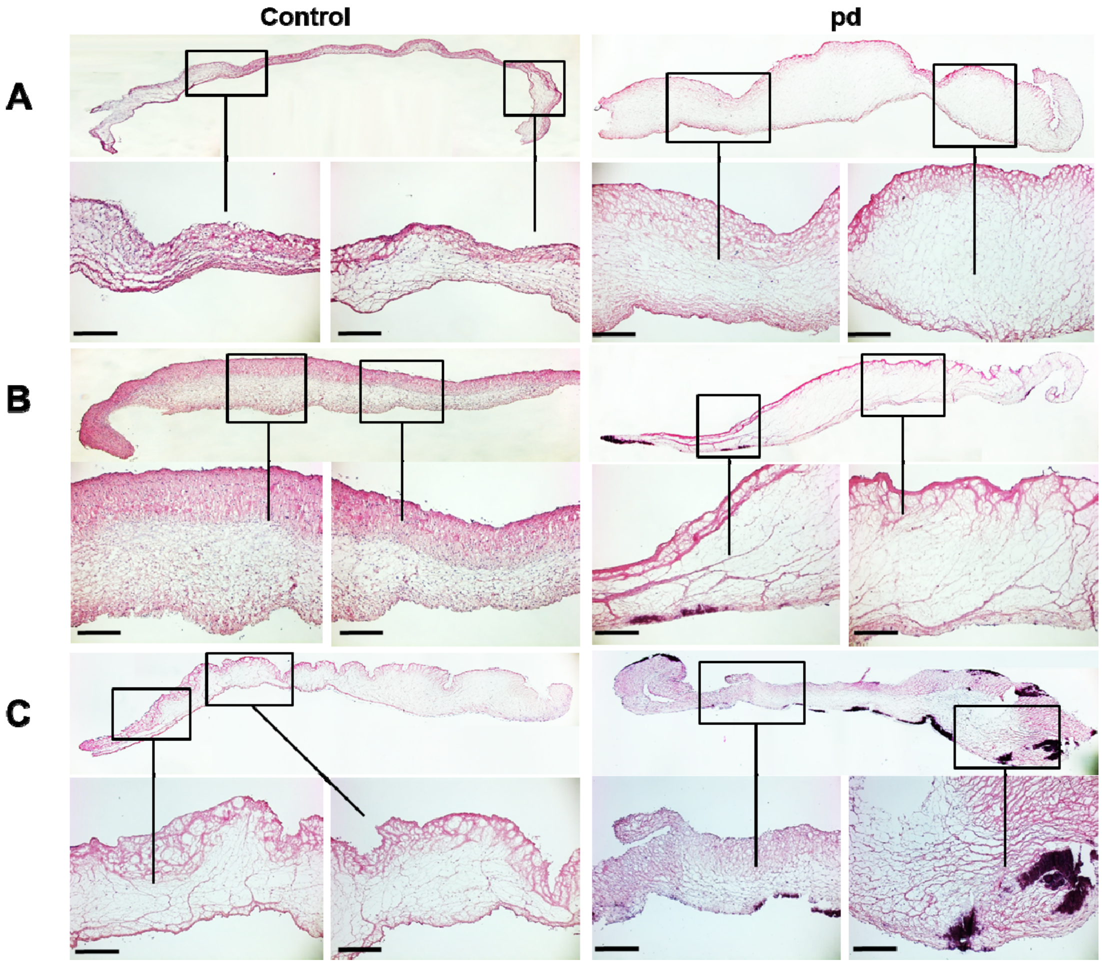

Figure S6. Temporal progression of ECM remodelling of AV leaflets. Haematoxylin-eosin (HE) staining of AV leaflets under pro-degenerative (pd) conditions (β-GP + $CaCl_2$) after 14 d (**A**), 28 d (**B**) and 56 d (**C**) compared to control conditions. Scale bar indicates 100 µm. Representative images of five different experiments are shown.

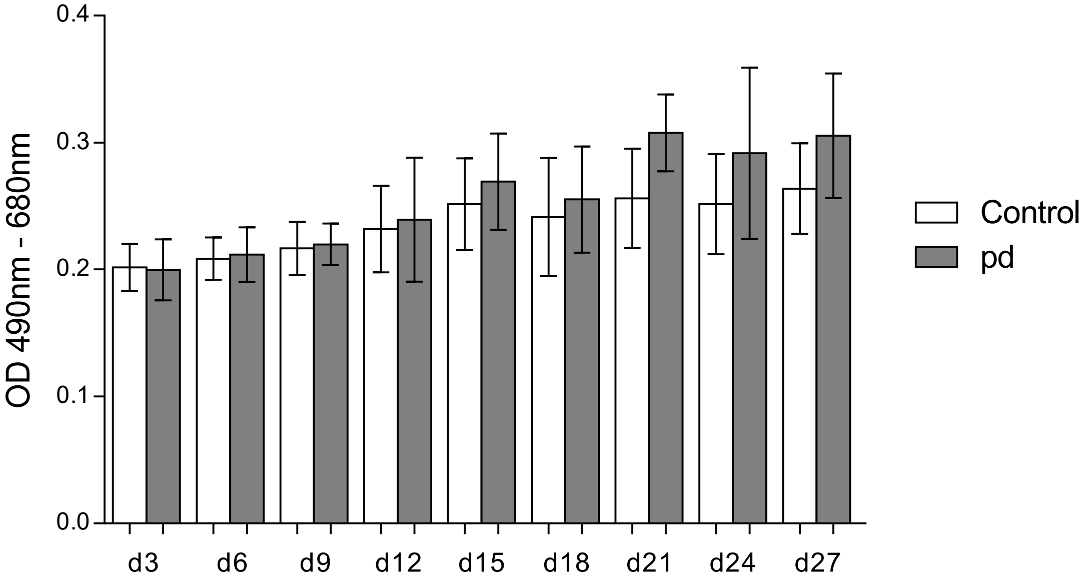

Figure S7. Analysis of LDH secretion. Analysis of LDH levels in supernatants (*n* = 5) of AV tissue cultures under control (white column) and under pd-conditions (grey column).

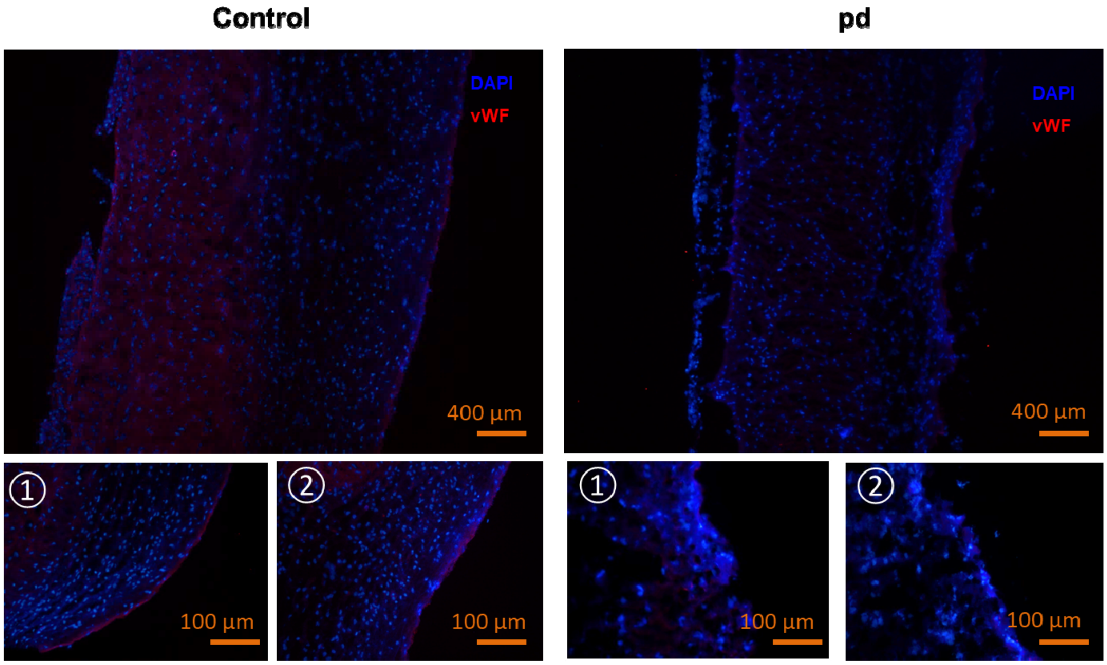

Figure S8. Analysis of endothelial layer. Immunohistological images with antibodies against von Willebrand factor (vWF) of AV leaflets under pro-degenerative (pd, β-GP + $CaCl_2$) and control conditions. One overview and two different enlargements (① and ②) are shown. Scale bar indicates 400 or 100 µm.

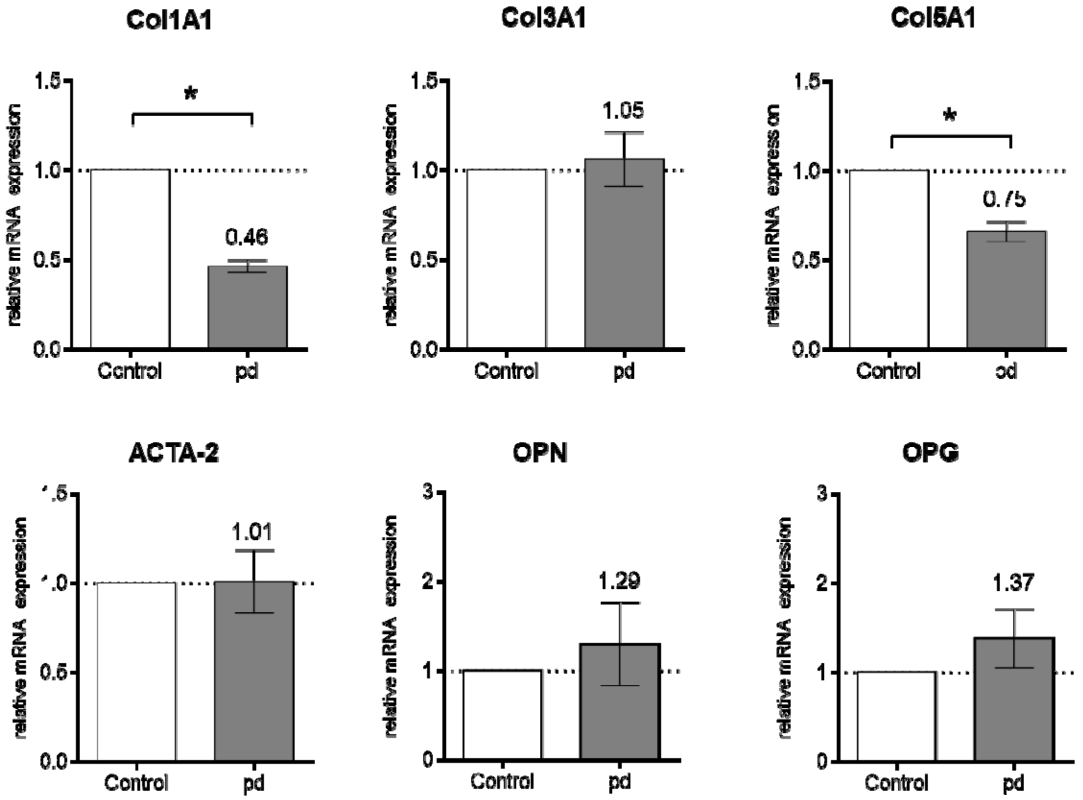

Figure S9. Gene expression analysis of AV leaflets after 14 d. Analysis of gene expression of AV leaflets (dark column, 14 d) under pro-degenerative (pd) conditions for alpha-1 type I collagen (Col1A1), alpha-1 type III collagen (Col3A1), alpha-1 type V collagen (Col5A1), alpha smooth muscle actin (ACTA2), osteopontin (OPN) and osteoprotergerin (OPG) compared to control conditions (white column). Data (n = 4) are mean ± SEM. P-values are calculated by using Kruskal-Wallis test with Dunn's multiple comparison post hoc test. * $p < 0.05$.

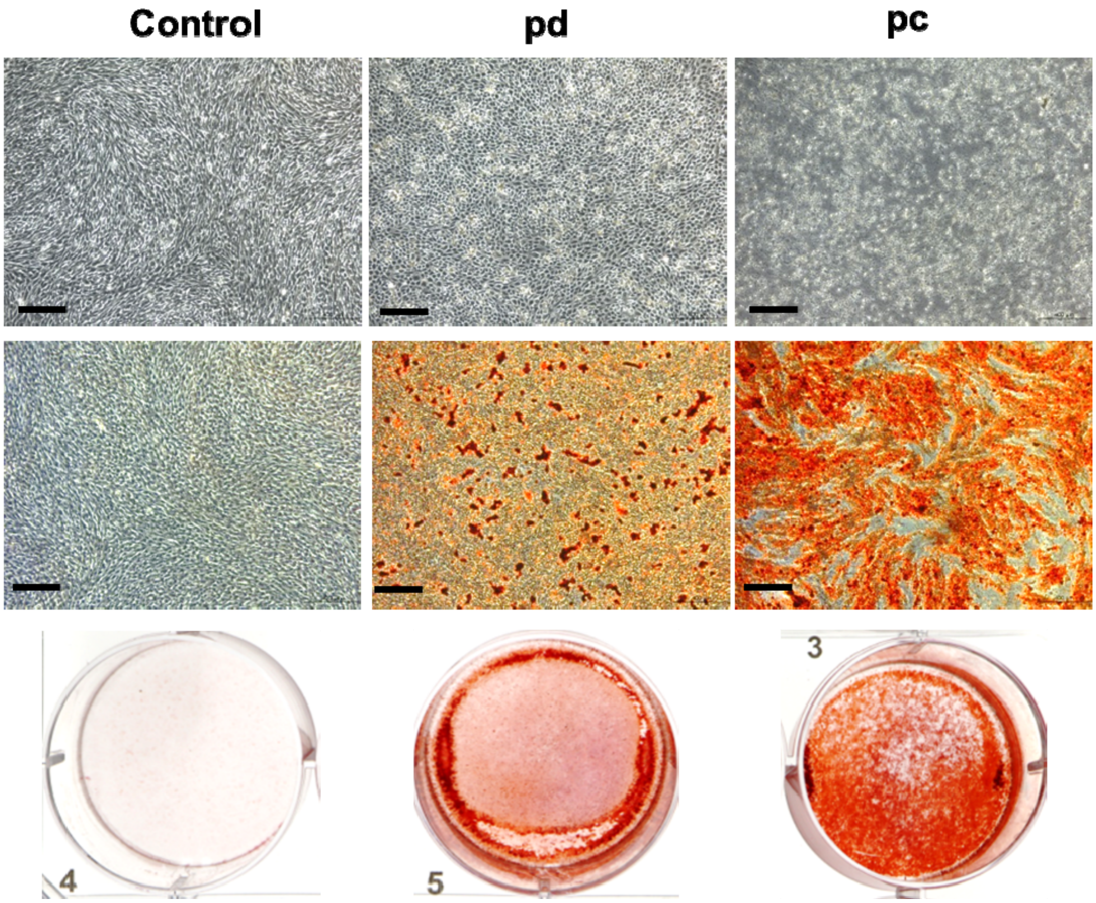

Figure S10. Comparison of pro-degenerative and pro-calcifying conditions. Light microscopy images and alizarin red S staining of VIC cultures (*n* = 16) after 7 d under pro-degenerative (pd) and pro-calcifying (pc) conditions. Scale bar indicates 400 µm.

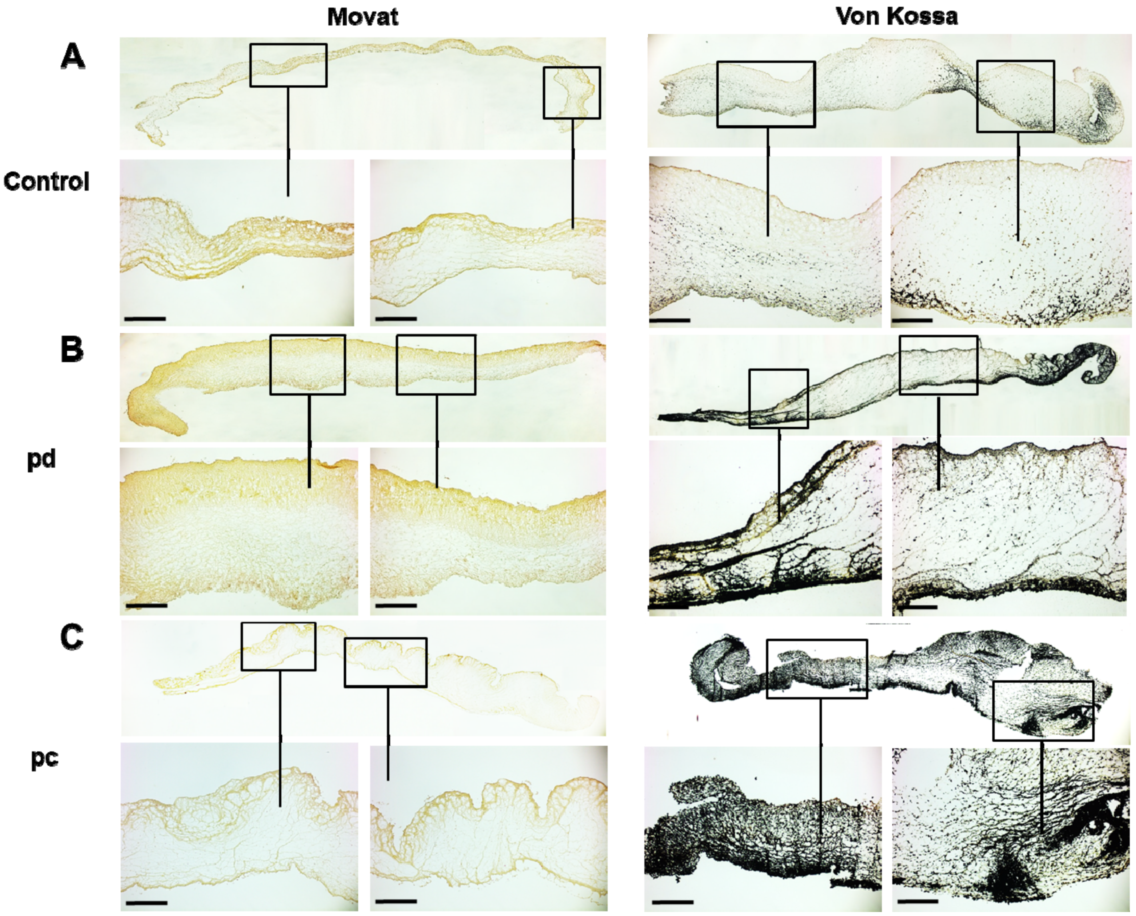

Figure S11. Temporal progression of AV leaflet degeneration. Von Kossa staining of AV leaflets after 28 d under pro-degenerative (β-GP + $CaCl_2$, **B**) and pro-calcifying (pc, NaH_2PO_4, **C**) compared to control conditions (**A**). Scale bar indicates 100 µm. Representative images of five different experiments are shown.
